# Perceived Parenting Styles of Individuals With Gender Dysphoria

**DOI:** 10.3389/fpsyg.2021.655407

**Published:** 2021-11-11

**Authors:** Cláudia C. Garcia, Karine Schwarz, Angelo B. Costa, Cesar A. Bridi Filho, Maria Inês R. Lobato

**Affiliations:** ^1^Programa de Pós-graduação em Psiquiatria e Ciências do Comportamento, Universidade Federal do Rio Grande do Sul, Porto Alegre, Brazil; ^2^Fundação de Amparo à Pesquisa do Estado do Rio Grande do Sul, Porto Alegre, Brazil; ^3^Postgraduate Program in Psychology, Pontifical Catholic University of Rio Grande do Sul, Porto Alegre, Brazil; ^4^Clinical Hospital of Porto Alegre, Porto Alegre, Brazil

**Keywords:** transgender, gender identity, family, gender dysphoria, neglect

## Abstract

**Objective:** To map patterns of behavior of parents and/or caregivers as perceived by their adult children, transgender patients seen through the Programa Transdisciplinar de Identidade de Gênero, and to determine if one parenting style was more prevalent.

**Design:** 82 patients were interviewed by the Parenting Style Inventory.

**Results:** The 82 patients (32 transgender men and 50 transgender women) completed a total of 145 protocols, being 65 concerning their fathers, and 80 concerning their mothers. The transgender women’s perceptions of their mothers were significantly different from those concerning their fathers. The transgender men and women had a positive mean perception of their relationship with their mothers and a negative mean perception of their fathers. The transgender women had on average a positive perception of their relationship with their mothers and a negative perception of their relationship with their fathers. This difference in perception was primarily in positive practices; the women felt that their mothers exhibited more positive practices of Positive Monitoring (A) and Moral Behavior (B) than their fathers. When we compared negative practices, negligence alone was considered the worst parental pattern by both transgender men and women.

**Conclusion:** Our study shows that fathers, more so than mothers, need to be encouraged to participate in the process of understanding the transgender condition and that in general, families need to be supported by mental health professionals to provide a more welcoming environment for individuals with Gender Dysphoria.

## Introduction

A recent publication by the World Health Organization (WHO) in June 2018 announced a new edition of the International Classification of Diseases, the ICD-11 (the new version will be presented for adoption by the Member States in May 2019 and will come into use January 1, 2022). In this new edition, the WHO has removed transgender from the mental illness chapter and renamed it gender incongruity. The 5th edition of the Diagnostic and Statistical Manual of Mental Disorders—the DSM-5 (American Psychiatric Association, 2013)—uses the term gender dysphoria, which has the following diagnostic criteria: marked incongruity between the experienced/expressed gender and the gender assigned at birth; strong desire to get rid of primary and/or secondary sex characteristics because of the marked gender incongruity; duration of at least 6 months; and association with impairment in social, academic or other important areas of the individual’s life that may cooccur with sexual development disorders. The estimated prevalence of gender dysphoria for males at birth ranges from 0.005 to 0.014%; for females at birth, it ranges from 0.02 to 0.03% (American Psychiatric Association, 2013).

According to Weber et al. (2004), parents influence the behavioral, emotional, and intellectual development of their children through parent-child interactions. Patterson (1982) emphasizes that practices such as setting of rules and limits, and affective involvement may be associated with good child development. By contrast, authoritarian practices such as strict discipline can lead to maladaptive social behaviors as well as anxiety and depression.

Mondim (2008) notes that parents whose educational practices are based on problem solving and the use of appropriate, calm, and peaceful strategies and dialogues are more likely to have children who develop characteristics such as respect and confidence and who solve their problems without using aggression. Gomide (2006) emphasizes that the construction of the child is based on a model of conduct and moral standards set by the parents. Rules must be firm and clear but not threatening, as threats can lead to insecure, emotionally manipulative and/or aggressive individuals. The physical and moral integrity of the child should be paramount for parents and caregivers. Parents have a responsibility to create a connection between the family’s internal world and the external social world by establishing moral rules and providing opportunities for learning.

Parenting style has been defined by several authors, each of whom broadened the focus to develop their research. Darling and Steinberg (1993) describe parenting style as a set of specific educational practices that allow the assessment of parents and/or caregivers’ influence on their children’s expressed behaviors, be they normative or maladaptive. These authors point out that since the 1930s, research related to this topic has been the object of investigation by several researchers.

In Baumrind (1966) model, the adopted parenting practices serve as a foundation for research on parent-child education, guiding the concepts of parenting styles, and integrating emotional and behavioral aspects. The author asserts that the authoritative model (the presence of established rules and limits, but in a non-repressive, participatory fashion) is more effective than the two other types of control: authoritarian (demanding and controlling) and permissive (low demands).

Parental representations of transgender women and men in relation to control groups matched for age, educational level and number of siblings were initially assessed by Cohen-Kettenis and Arrindel (1990) using the Inventory for Assessing Memories of Parental Rearing Behavior (EMBU). Results showed that transgender men rated both (fathers and mothers) as more rejecting and less emotionally affectionate, but only their mothers as more protective than their rated female control counterparts. Furthermore, transgender men and women differed from each other in some aspects (lower scores on parental emotional warmth and higher scores on maternal rejection for female patients).

**FIGURE 1:**
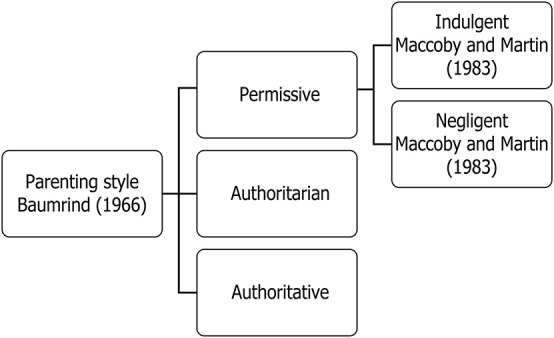
Parenting style as presented by Baumrind (1966) and modified by Maccoby and Martin (1983).

In the early 1980s, however, Maccoby and Martin (1983) suggested two parental educational practices: demand and responsiveness. The first refers to how parents control their children’s behavior with limits and rules. The second refers to the type of emotional support that parents favor, which influences their children’s development and autonomy. The authors later promoted the splitting of the permissive parenting style into two other concepts: indulgence (in which the child monitors his or her own behavior) and negligence (in which the parent has little or no involvement in the child’s behavior), as shown in Figure 1.

**FIGURE 2:**
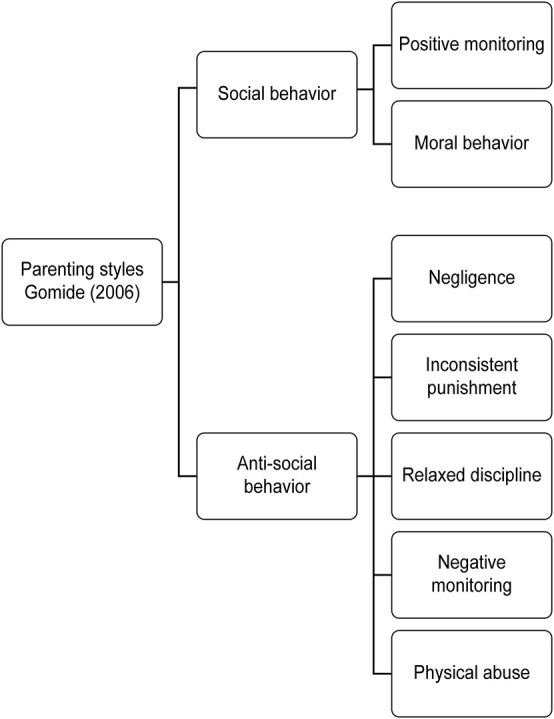
Parental styles (Gomide, 2006).

The Parental Style Inventory (PSI) is divided into two specific types of behaviors: social (positive) behaviors and antisocial (negative) behaviors, as shown in Figure 2 (Gomide, 2006). Positive social behaviors include positive monitoring (i.e., knowing one’s children’s whereabouts and activities and how they adapt) and moral behavior (i.e., imparting values such as justice, compassion, and friendship), which contribute to better self-esteem and autonomy; in contrast, antisocial-negative behaviors include inconsistent punishment, neglect (i.e., ignoring one’s children’s behavior and providing little response when they initiate communication), relaxed discipline (i.e., withdrawing from conflict when children do not comply with established rules), negative monitoring (i.e., excessive control over and orders given to children), and physical abuse (i.e., the use of physical force with the intention of causing pain), which can lead to aggressive behavior, low self-esteem, poor tolerance for frustration, difficulty following social norms and lack of regret.

The objective of this study was to characterize the family model of patients with GD from the patients’ perspective and to identify whether the perspectives differed between GD groups (transgender women and transgender men). For this purpose, the PSI was used (Gomide, 2006).

## Materials and Methods

### Participants

To calculate the sample size we used the WinPepi program, version 11.65. Considering a confidence of 95%, a margin of error of two units and a standard deviation of 10.02 in the children’s IEP scale, as mentioned in Sampaio and Gomide (2007) the sample size was 99 participants. An average of two acceptable error points was considered for this study, since clinical practice with this population manifests aspects of family absence, abandonment, maltreatment, negligence, among others.

The individuals with gender dysphoria (GD) who participated in this study and were seen at the outpatient clinic of the *Programa Transdisciplinar de Identidade de Gênero* (Gender Identity Transdisciplinary Program; PROTIG) at the *Hospital de Clínicas de Porto Alegre* (Porto Alegre General Hospital; HCPA) reported that they came from families with a history of physical violence, abandonment, and emotional neglect.

All adult patients were invited to participate and those who expressed their agreement through the Informed Consent Form were included in the survey. Of the 94 adult patients seen by PROTIG, monthly, 82 subjects (32 transgender men and 50 transgender women) between 18 and 59 years of age accepted to participate in the study. Of the 82 participants, 38 were 29 years old or younger, 29 were between 30 and 39 years of age, and 13 were over 40 years old. Regarding education level, 51 had a high school education or less, and 29 had begun or completed higher education. A sociodemographic characterization of the sample is shown in Table 1.

**TABLE 1:** Sociodemographic characterization of the sample.

**Feature**	**n (%) (*N* = 82)**
**Age group**	
Up to 29 years	38 (46.3)
30–39 years	29 (35.4)
Over 40 years	13 (15.9)
Did not answer	2 (2.4)
**Gender**	
Transgender men	32 (39.0)
Transgender women	50 (61.0)
**Education level**	
High school or less	1 (0.6)
Began or completed higher education	2 (1.1)
Did not answer	19 (10.7)

### Procedures

This was a cross-sectional study that used convenience sampling at PROTIG/HCPA from October 2017 to July 2018. The aim was to characterize the family model of patients with GD seen at the outpatient clinic of PROTIG/HCPA (approved project 2018/0201) based on their insights and using the PSI scale. The inclusion criteria were as follows: patients seen at the PROTIG outpatient clinic with a diagnosis of GD according to the DSM-5 (American Psychiatric Association, 2013). The data were collected in person on the HCPA premises. The participants were invited to participate by the lead researcher, and, prior to completing the questionnaire, the individuals expressed their willingness to participate by signing an informed consent form. Anonymity was guaranteed, and only the researchers had access to the data as required in the ethical considerations of Resolution n. 510/2016 of *Conselho Nacional de Saúde* (Brazilian National Health Council) for research on human beings.

### Instruments

The complete survey was composed of a sociodemographic questionnaire and the PSI (Gomide, 2006).

### Parenting Style Inventory

This instrument consists of 42 items that correspond to seven educational practices. For each educational practice, six questions were formulated and distributed throughout the inventory. Of the seven educational practices, two correspond to positive educational practices: (A) Positive monitoring and (B) moral behavior, and five refer to negative educational practices: (C) inconsistent punishment, (D) negligence, (E) relaxed discipline, (F) negative monitoring, and (G) physical abuse. The Parenting Style Index (PSIs) is calculated by adding the positive practices (A + B) and the negative practices (C + D + E + F + G) and then subtracting the sum of the negative practices from the sum of the positive practices: PSI = (A + B)-(C + D + E + F + G), according to the author’s recommendation. When the parental index is positive, it indicates a strong presence of positive parenting practices (positive monitoring and moral behavior) that outweighs negative practices (inconsistent punishment, neglect, relaxed discipline, negative monitoring and physical abuse).

Scores on the PSI can vary from -60 (complete absence of positive practices and exclusive presence of negative practices) to + 24 (absence of negative practices and exclusive presence of positive practices). The score result is categorized into four categories: (1) Great parenting style: no negative parenting practices; (2) Regular parenting style above average and (3) Regular below-average parenting style: both include variations of positive and negative practices; and (4) Risky Parenting Style: a predominant use of negative practices to the detriment of the positives.

### Data Analysis

First, Cronbach’s alpha for the PSI was calculated to investigate its internal consistency. Then, the participants were divided into three groups based on their age: up to 29 years old, from 30 to 39, and over 40 years old. An analysis of variance (ANOVA) was then performed between the PSI scores and the age ranges.

## Results

Each participant responded to one protocol concerning their mother and/or another concerning their father; therefore, some completed a protocol for only one parent seeing as they had not been raised by both parents. Thus, a total of 145 protocols were completed—65 concerning fathers and 80 concerning mothers. Cronbach’s alpha for the PSI was 0.77, which indicates reasonable internal consistency of the questionnaire according to Pestana and Gageiro (2008). Table 2 shows the means and standard deviations of the Maternal and Paternal PSIs based on the respondents’ perceptions. Only the maternal perceptions of the female participants obtained a positive mean value; the others obtained negative means. Table 3 shows the means for each protocol.

**TABLE 2:** Means and standard deviations of parenting style indices.

**Index**	**N (samples)**	**Mean**	**Standard deviation**	**Minimum**	**Maximum**
Maternal PSI_TM	30	–2.87	12.41	–27	15
Paternal PSI_TM	27	–4.52	11.27	–30	11
Maternal PSI_TW	50	1.92	11.62	–27	21
Paternal PSI_TW	38	–5.82	14.20	–38	16

*TM, Transgender men; TW, Transgender women.*

**TABLE 3:** Average and standard deviation of maternal and paternal (PSI).

	**N (samples)**	**A**	**B**	**C**	**D**	**E**	**F**	**G**
Transgender men—maternal	30	7.47^a,b^ (3.44)	7.83^a,b^ (2.20)	3.57 (2.40)	4.60^a,b^ (3.40)	2.97 (2.63)	5.03^a^ (2.34)	2.00 (2.00)
Transgender men—paternal	27	5.56^b^ (3.14)	6.81^b^ (3.34)	3.04 (2.44)	5.30^a^ (3.45)	2.26 (1.91)	3.48^a^ (2.24)	2.81 (3.64)
Transgender women—maternal	50	9.08^a^ (2.47)	9.46^a^ (2.34)	3.48 (2.44)	3.06^b^ (2.97)	2.92 (2.11)	5.00^a^ (2.09)	2.16 (2.90)
Transgender women—paternal	38	5.61^b^ (3.58)	7.31^b^ (3.32)	3.87 (2.93)	4.76^a,b^ (3.63)	2.66 (2.10)	3.87^a^ (3.07)	3.58 (3.78)

*^*a,b*^Means followed by different letters within a column are significantly different from each other according to Tukey’s test.*

When the perceptions of transgender women regarding maternal and paternal parenting styles were analyzed separately from those of transgender men, it was possible to observe that the women perceived the maternal parental style with a positive value on average, characterizing a regular parenting style. The transgender women perceived the paternal parenting style as negative, corresponding to a style that offers risk. The transgender men perceived both maternal and paternal parenting styles as negative, both corresponding to risky parenting styles.

“The results described in Table 3 indicate that transgender women’s perceptions of their mothers were significantly different from those concerning their father (*p* = 0.001, *t* = 3.77, *df* = 37, and Cohen’s *d* = 0.60).”

The transgender women had on average a positive perception of their relationship with their mothers and a negative perception of their relationship with their fathers. This difference in perception was primarily in the area of positive practices; the women felt that their mothers exhibited more positive practices (practices A and B in the questionnaire) than their fathers (*p* = 0.0001, *F* = 12.11, MS = 116.15, *df*(factor) = 3, *df*(error) = 141; *p* = 0.0001, *F* = 6.90, MS = 54.15, *df*(factor) = 3, *df*(error) = 141, respectively).

A *post hoc* analysis using Tukey’s HSD (see Table 3) showed that for practice A, the maternal mean did not differ between the transgender women and transgender men, but it did differ from and have a higher value than the paternal mean for both transgender men and transgender women. The same was true for practice B, showing that the mothers of the transgender women were perceived to exhibit more positive behaviors than the fathers of both the transgender men and the transgender women.

For practice D, the paternal mean for the transgender men was higher than the maternal mean for the transgender women, showing that the fathers of transgender men were perceived to exhibit more negative behaviors than the mothers of transgender women [*p* = 0.019, *F* = 3.43, MS = 38.0, *df*(factor) = 3, *df*(error) = 141]. The other practices, C-E-F-G, did not differ between categories [*p* = 0.644, *F* = 0.56, MS = 3.69, *df*(factor) = 3, *df*(error) = 141; *p* = 0.571, *F* = 0.67, MS = 3.22, *df*(factor) = 3, *df*(error) = 141; *p* = 0.017, *F* = 3.50, MS = 21.15, *df*(factor) = 3, *df*(error) = 141; *p* = 0.152, *F* = 1.79, MS = 19.4, *df*(factor) = 3, *df*(error) = 141, respectively].

Other analyses identified a significant difference (*p* = 0.003, *t* = 3.10, *df* = 63) between the interviewees’ perceptions of their fathers compared to their perceptions of their mothers (with no difference between the transgender men and the transgender women). These results showed that transgender men and women had a positive mean perception of their relationship with their mothers (mean = 0.13) and a negative mean perception of their fathers (mean = –5.28), as shown in Table 4.

**TABLE 4:** Negligence: Mean perception of parental style (PSI).

	**N (samples)**	**Mean**	**Standard deviation**	**Minimum**	**Maximum**
Maternal PSI_	80	0.13	12.07	–27	21
Paternal PSI_	65	–5.28	12.98	–38	16

## Discussion

There is a well-known and generalized history of rejection and mistreatment of people with GD, which is described as a feeling of incongruity between one’s gender identity and the sex assigned at birth (Lai et al., 2010). In one of the pioneering studies on parenting styles, the authors highlighted that transgender man rated their parents as less affectionate, more rejecting and more protective than male controls rated their parents (Cohen-Kettenis and Arrindel, 1990). This history has served as an impetus for research and has motivated a worldwide task force fostered by the World Professional Association for Transgender Health (WPATH), with the goal of educating and supporting family members of individuals with GD in attempts to minimize psychiatric and social morbidity resulting from abandonment and family rejection. The maternal/paternal role within the nuclear family may vary in different cultures, but it seems reasonable to infer that Brazilian society may be similar to that of other Western countries.

In our society, according to the latest census of the IBGE/2010, the number of households with a female head of family has increased considerably, rising from 22.2% in 2000 to 37.3% in 2010. These data include broken families, in which increased economic and childcare responsibility rests on the mother. In this sense, and considering sociocultural differences, homophobic feelings are still present in our society, and fathers have more difficulty than mothers dealing with children with gender issues, especially transgender women. Our findings agree with this data; however, since this study did not involve qualitative interviews and there was a small number of participants and no control group, it is not possible to make affirmations in that regard—additional research remains to be done in later studies. Moreover, mothers are more likely than fathers to accept variations in terms of gender and sexual orientation, either due to subjective issues related to the social identity of motherhood or due to the mother-child bond. This agrees with previous studies which show that fathers of transgender are my hostile than those of a control group, while mothers tend to be perceived as more intrusive, as seen in a study by Simon et al. (2011). Lai et al. (2010), however, found opposing results in their study, which showed that adults with gender dysphoria perceived less support and affection and more authoritarian control from both their parents in comparison to those without GD. Since their study was not conducted in a western society, it is possible that may be the cause of this difference.

In our study, the age and education level of the participants did not influence their PSI scores. Therefore, we did not find generational differences when we examined the hypothesis that there would be a more positive parenting style among those born after the 1990s, as we anticipated that they would have been educated in a more tolerant environment than previous generations.

Considering the abovementioned maternal context, it is not surprising that our patients, both transgender men and transgender women, generally showed a healthier bond with their mothers and felt that their mothers used more positive educational practices than their fathers.

The results also demonstrate that transgender women perceive their mothers significantly differently from how they perceive their fathers. The transgender women in our study had a positive mean perception of their relationship with their mothers and a negative mean perception of their relationships with their fathers, thereby reinforcing the idea of the positive importance of the maternal role in the family constellation. This is in agreement with previous studies, as shown by Lai et al. (2010).

When comparing the study groups, we found a more positive attitude among the mothers of transgender women than among the mothers of transgender men. This result is in accordance to that of the study conducted by Simon et al. (2011), which reported that transgender men experience less maternal care than transgender women or non-transgender women. It is possible that gender identification facilitates dialogue between daughters and mothers. Transgender women need to “learn” to be women. According to Simon et al. (2011), transgender women are made to feel by their mothers as defective. The mothers of the transgender men had lower scores for positive attitudes. It can be inferred that both the mothers and their children (transgender men) mutually rejected each other for reasons that did not apply to the transgender women (as they had no identity to be imitated or learned from their mothers). It is also possible that mothers of transgender men feel “more guilt” for their “daughters” lack of femininity, which they interpret as a (maternal) flaw in the process of developing their children’s personalities, which may lead to frustration, mutual demands and resentment.

Another result that confirms the difference between mothers’ and father’s views concerns the practice of negligence (practice D). The transgender men viewed their fathers as being more negligent (*p* = 0.007; *t* = 2.85) than the transgender women viewed their mothers. Paternal neglect among transgender men may be related to a general cultural disinterest in the outcomes of women (even daughters) and, therefore, a greater interest in males at birth. Another hypothesis is that “if he wants to be a man,” a transgender man needs to play the social roles expected for males (alleged emotional autonomy, proactivity, etc.). Another possibility is that fathers, unlike mothers, feel less responsible for outcomes in this area.

There were other results that we found interesting despite the absence of statistical significance. The mothers had higher score for negative monitoring (practice F) for both the transgender men and the transgender women. This type of monitoring suggests attitudes that are meant to protect but are excessively controlling, which likely reflects anxiety and a sense of helplessness in dealing with the educational needs of their children. Thus, the mothers used resources such as oversight and excessive orders. Another result points to greater use of physical abuse by the fathers of transgender women. These data are in agreement with the observation that fathers are less tolerant of variations in sexual orientation and gender when compared to mothers and that the use of violent “corrective” methods may reflect the fantasy that “fear” can prevent the development of GD. However, both negative monitoring and physical abuse represent feelings of anger and frustration with GD and the likely attempt to deal with feelings of impotence in relation to this situation. Despite cultural and trans-generational differences, we all tend to repeat familial educational models, and the use of physical punishment as an educational tool has only recently been called into question and forbidden in Brazilian society.

It is important to emphasize that the descriptive nature of this study prevents us from making extrapolations to other populations besides the study, transgender people who seek gender affirmation surgeries. Furthermore, the fact that we do not have a paired control group prevents us to extrapolate these results. The lack of a control group, as a limitation, means that the data may simply indicate that, regardless of whether their children are transgender, Brazilian parents may have the characteristics presented here. Nevertheless, given that the theme of this study is little explored in the literature, future works can address these limitations with a more comprehensive sample of the transgender community and a control group.

## Conclusion

Since 1998, individuals who experience a discrepancy between the gender assigned to them at birth and their expressed identity have found support in PROTIG/HCPA, an ally in helping transgender individuals who wish to undergo gender reassignment surgery and those who achieve this milestone. PROTIG/HCPA is also a promoter of understanding GD through research.

Positive family relationships are associated with better interpersonal relationships, quality of life and emotional balance. In the context of transgenderism, where there are incredible social and affective barriers, the family is the foundation for better social adaptation. Our study shows that fathers, more so than mothers, need to be encouraged to participate in the process of understanding the transgender condition and that in general, families need to be supported by mental health professionals to provide a more welcoming environment for individuals with GD. This study sample should be expanded upon, and parents’ own perceptions should also be included. Further studies could also include qualitative interviews and a control group.

## Data Availability Statement

The original contributions presented in the study are included in the article/supplementary material, further inquiries can be directed to the corresponding author/s.

## Ethics Statement

The studies involving human participants were reviewed and approved by Comitê de Ética em Pesquisa—HCPA. The patients/participants provided their written informed consent to participate in this study.

## Author Contributions

CG: data collection, idea, and article writing. KS, AC, and CB: final review and formatting. ML: idea and article writing. All authors contributed to the article and approved the submitted version.

## Conflict of Interest

The authors declare that the research was conducted in the absence of any commercial or financial relationships that could be construed as a potential conflict of interest.

## Publisher’s Note

All claims expressed in this article are solely those of the authors and do not necessarily represent those of their affiliated organizations, or those of the publisher, the editors and the reviewers. Any product that may be evaluated in this article, or claim that may be made by its manufacturer, is not guaranteed or endorsed by the publisher.
